# Women’s and maternity care providers’ perceptions of pain management during childbirth in hospitals in Southern Tanzania

**DOI:** 10.1186/s12884-024-06606-9

**Published:** 2024-06-10

**Authors:** Katrine Thorgaard-Rasmussen, Helle Mölsted Alvesson, Andrea B. Pembe, Lilian T. Mselle, Regine Unkels, Emmy Metta, Fadhlun M. Alwy Al-beity

**Affiliations:** 1 Department of Global Public Health, Karolinska Institutet, Stockholm, Sweden; 2 Department of Obstetrics and Gynaecology, Muhimbili University of Health and Allied Sciences, Dar es Salaam, Tanzania; 3 Department of Clinical Nursing, School of Nursing, Muhimbili University of Health and Allied Sciences, Dar es Salaam, Tanzania; 4 Department of Behavioural Sciences, School of Public Health and Social Sciences, Muhimbili University of Health and Allied Sciences, Dar es Salaam, Tanzania

**Keywords:** Pain management, Tanzania, Maternity care providers, Labour pain, Childbirth, Midwifery

## Abstract

**Background:**

The majority of women experience pain during childbirth. Offering and supporting women to use different methods for coping with pain is an essential competency for maternity care providers globally. Research suggests a gap between what women desire for pain management and what is available and provided in many low-and middle-income settings. The study aimed to understand how pain management is perceived by those involved: women experiencing childbirth and maternity care providers.

**Methods:**

Individual semi-structured interviews with women (*n* = 23), maternity care providers (*n* = 17) and focus group discussions (*n* = 4) with both providers and women were conducted in two hospitals in Southern Tanzania in 2021. Transcribed interviews were analysed using reflexive thematic analysis. Coding and analysis were supported by the software MAXQDA.

**Results:**

Three main themes were generated from the data. The first, ‘pain management is multifaceted’, describes how some providers and women perceived pain management as entailing various methods to manage pain. Providers perceived themselves as having a role in utilization of pain management to varying degree. The second theme ‘pain management is primarily a woman’s task’ highlights a perception of pain management as unnecessary, which appeared to link with some providers’ perceptions of pain as natural and necessary for successful childbirth. Few women explicitly shared this perception. The third theme ‘practice of pain management can be improved’ illustrates how women and maternity care providers perceived current practices of pain management as suboptimal. According to providers, this is primarily due to contextual factors such as shortage of staff and poor ward infrastructure.

**Conclusion:**

Women’s and maternity care providers’ perceptions ranged from perceiving pain management as involving a combination of physiological, psychological and social aspects to perceive it as related with limited to no pain relief and/or support. While some women and providers had similar perceptions about pain management, other women also reported a dissonance between what they experienced and what they would have preferred. Efforts should be made to increase women’s access to respectful pain management in Tanzania.

**Supplementary Information:**

The online version contains supplementary material available at 10.1186/s12884-024-06606-9.

## Background

The majority of women experience pain during childbirth and for many it is the most severe pain they experience in their life [1, 2]. Unlike other acute or chronic pain experiences, labour pain is not associated with pathology and while some women describe their experience as excruciating, others refer to labour pain as purposeful, positive and physiological [2–4]. Women’s experiences of pain are subjective and complex and are influenced by physiological causes (e.g. intensity of contractions, dilation of the cervix etc.), psychological factors (e.g. ascribed meaning to pain, fear and anxiety) and the social environment surrounding them (e.g. maternity care provider, companion) [1–2, 5–8]. As labour pain is complex and unique, it can consequently be complex to manage [1, 3].

The World Health Organization (WHO) highlights pain management as a component of good quality care, that should be offered to all women globally [9]. Offering and supporting women to use different methods for coping with labour pain is defined as an essential competency for midwives by the International Confederation of Midwives [10]. Some women cope with pain without any intervention, but others require pain relief to get through labour [6, 11]. Appropriate management of pain can lead to women feeling safe, empowered and proud of their abilities and achievements [12]. In contrast, poor management of pain can affect women’s mental health and lead to women being fearful, which can affect how pain is experienced as well as women’s choices of place and mode of birth [7, 11].

Pain management encompasses different pain relief methods, but also other approaches to prevent and reduce pain, such as psychological support given by a maternity care provider or the presence of a companion during labour [6, 13]. Pain relief methods, such as pharmacological pain relief (e.g. epidural analgesia, opioids) and non-pharmacological pain relief (e.g. massage, breathing techniques, heat packs), are known as effective ways of reducing pain and associated with women reporting not only less pain, but also higher satisfaction [9]. Women’s experiences with both methods are, however, mixed which underlines the importance of supporting women’s individual choice of pain relief [9, 11]. Continuity of care as well as support from a provider or a companion are found to enhance women’s birth experiences, reduce the use of pain relief methods and are key contributors to women’s ability to cope with pain [8, 9, 14, 15].

Maternity care providers’ knowledge, skills, behaviour and attitude towards pain management are also known to influence pain management practices and women’s satisfaction with their birth experiences [15, 16]. Leap and Hunter described two distinct, but overlapping approaches to pain management that are commonly adopted by maternity care providers [4]. Firstly, the ‘*pain relief’-approach* where providers frequently offer pharmacological pain relief in order to minimize discomfort and ensure ‘adequate’ relief. Secondly, the ‘*working with pain’ approach* where the provider believes that the woman can cope with pain when receiving appropriate support. Both approaches can affect how women see themselves and their abilities, thus affecting their experiences of childbirth [1, 4].

Pain relief methods are frequently utilized in high-income settings [9]. Studies from many low-and-middle income countries (LMICs), however, indicate that even though many women desire pain relief during childbirth, availability of pharmacological pain relief is low and the practice of pain management poor [11, 17–24]. Recent studies from Tanzania have found that although maternity care providers demonstrated some knowledge of pain relief methods, they did not offer or utilize these routinely [20, 22]. Furthermore, it is commonly reported, that women in LMICs experience disrespectful treatment from healthcare providers during childbirth [25–29]. To improve pain management for women, we must first improve our understanding of how pain management is perceived by those involved. In Tanzania, research on maternity care providers’ perceptions of pain management is limited and while women’s experiences are described, reports on their perceptions of pain management are equally scarce [22]. Therefore, the aim of this study was to understand how women and maternity care providers perceive pain management during childbirth in hospitals in Southern Tanzania.

## Methods

### Study design

This was a qualitative study grounded in constructivism and nested within the larger research project ***A****ction ****L****everaging ****E****vidence to ****R****educe perinatal Mor****t****ality and morbidity* (ALERT), which aims to develop and evaluate intrapartum quality improvement interventions in Benin, Malawi, Uganda and Tanzania [30]. This study was part of the initial co-design phase of the ALERT project in Tanzania, where the end-user’s perspectives were explored and integrated in an intervention design. We used individual semi-structured interviews and focus group discussions (FGDs) to capture women’s and maternity care providers’ perceptions on pain management. Maternity care providers include nurses, nurse-midwives, midwives, auxiliary staff, associate clinicians and medical doctors [30].

### Study setting

The health care system in Tanzania is decentralized and 70% of all health facilities are publicly owned and managed [31]. According to health policy, there are no official user fees for maternity care in facilities for women and children under five years [30, 32]. Informal payments, however, often for equipment and medicines, are commonly reported [24, 26, 28].

The workforce of maternity care providers in Tanzania encompasses different levels of staff training. Education of nurse-midwives in Tanzania progresses from certificate to diploma, degree and finally advanced practitioner, which reflects a differing number of years of training [33]. Similarly, it is common practice in Tanzania to have associate clinicians, such as assistant medical officers, working alongside university-trained medical doctors [34]. Assistant medical officers have fewer years of training, but are trained to perform emergency surgery, including caesarean Sect. [34].

Data were collected in two hospitals in two southern regions of Tanzania; one being a district hospital and another with a regional referral hospital status. The regions have on average a higher percentage of births assisted by skilled birth attendants than the country’s average (80% and 82% vs. the national average of 64%) [35]. The two hospitals were purposely selected to represent the two most commonly used facilities for childbirth in Tanzania: public and private-non-for-profit (faith-based) hospitals [30, 35]. Characteristics of both hospitals are listed in Table 1.

**Table 1:** Hospital characteristics (2021)

Hospital	Hospital 1	Hospital 2
Status	Public	Private non for profit
User fees	No	Yes
No. of births per year	2654	2532
No. of nurse/midwives in maternity ward	10	9
No. of medical doctors in maternity ward	2 + medical interns^1^	2
Obstetrician available	No	Yes

^1^There was one assistant medical officer working in the maternity wards during some of the study period

Women are usually accompanied or escorted by relatives to the hospitals. The escorting persons can be a family member, in-law, friend or neighbour. Upon arrival in the maternity ward, the escorting person stays outside while women enter. The escorting persons stay nearby (but out of the ward) for logistical purposes. In the labour wards, women are cared for by maternity care providers until birth. After an uncomplicated vaginal birth, women are observed for two hours in the labour ward and transferred to the postnatal ward for 24 h observation before discharge. Women who develop complications are reviewed by medical doctors for decision-making and surgery. Women who give birth by caesarean section are usually discharged after 72 h.

With regard to pharmacological pain relief methods, both hospitals had access to paracetamol, diclofenac and some opioids, such as pethidine. Pain relief such as epidural analgesia was not available. Utilized pain relief methods in both hospitals, reported by women and maternity care providers, also included massage, breathing techniques and change of position during childbirth. Furthermore, women were encouraged to exercise, drink tea (to gain strength), empty the bladder frequently and take a bath when experiencing labour pain.

Both hospitals did not routinely allow birth companionship in the labour ward. The escorting persons were, however, within reach when needed to fetch medication, food or other logistical purposes.

### Data collection

Data were collected in two phases during 2021.

During the *first phase*, in February 2021, individual interviews were conducted with women and maternity care providers using topic guides informed by the literature on person-centred maternity care and WHO’s recommendations on intrapartum care (2018). The topic guides were tested during pilot interviews and adjustments were made accordingly. All participants were interviewed on several topics such as experiences of childbirth, communication, labour monitoring, privacy, newborn care and pain management. With regard to pain management, women were asked: “*When in pain, how were you supported/helped?”*, “*Who helped and supported you when you felt pain?”* and were further probed on, for example, if the received help and support made a difference to them and what they would have liked vs. what they actually received. They were further asked “*What pain relief options were offered, if any?”* and probed on acceptance, knowledge and communication. Maternity care providers were asked “*When women are in pain, what can you do to help them?”* and *“What pain relief options do midwives offer to mothers during labour, if any?”* They were probed on acceptance, preferred practices and support provided during labour pain.

The *second phase* of data collection was conducted in June 2021. As part of the co-design process in ALERT, data were analysed by the ALERT research team. Preliminary results from the interviews were discussed with women and maternity care providers in separate FGDs.

Nine data collectors with diverse professional backgrounds conducted all individual semi-structured interviews in phase one. Backgrounds included: obstetrics, midwifery, nursing, sociology and medical anthropology. All data collectors were fluent in Kiswahili, were familiar with the context and had previous experience with qualitative data collection. None of them were employed in any of the two hospitals. Two also conducted and facilitated the FGDs. During both data collection processes, the research team and team of data collectors had frequent discussions and reflection meetings.

Individual interviews and FGDs were conducted in a private setting within the hospitals. For hospital one, we used classrooms in the nursing school, which were within the hospital compound and for hospital two, we used vacant space in the new maternity building. All interviews and FGDs were conducted in Kiswahili and audio-recorded with the participants’ permission. The audio recorded interviews and discussions were transcribed verbatim and translated into English ensuring that non-Kiswahili-speaking researchers could participate in the analysis. Individual interviews lasted between 35 and 90 min and the FGDs lasted between 30 min and 2,5 h. In addition to the audio, field notes were taken for all interviews and FGDs and used during daily debriefing after data collection.

Data collection resulted in 23 individual interviews with women and 17 with maternity care providers and four FGDs were conducted, two with women (and their companions) (*n* = 12) and two with maternity care providers (*n* = 11). FGDs had five to six participants each. FGDs conducted with women also included their female companions, however, only the women’s point of view is reported in this study, making the number of participating women in the FGDs to be 7.

### Participant sampling, recruitment and characteristics

All participating women and maternity care providers in this study – in both phases of data collection – were purposively recruited. Women were recruited and interviewed within the first few days after birth, whilst still in the maternity ward. Participant’s in- and exclusion criteria, listed below, were the same for both individual interviews and FGDs. Sample size was assessed before, during and after data collection guided by the concept of information power by Kirsti Malterud et al. [36]. They suggested that sample size should be guided by (a) the aim of the study (b) sample specificity (c) use of established theory (d) quality of dialogue and (e) analysis strategy. In order to obtain broad representation of both women and maternity care providers, a relatively large sample size was needed.

*All women* who had given birth to a newborn with a birthweight ≥ 1000 g. (considered proxy for viability) in the two hospitals, were eligible for participation [30]. Women were sampled based on maximum variation of mode of birth/outcome (uncomplicated vaginal birth, caesarean section, complications (e.g. vacuum extraction, premature baby)). Women with antepartum fetal death were excluded. Medical personnel from included hospitals who gave birth at the time of data collection were excluded.

All professionally trained *maternity care providers*, working in the two hospitals, were eligible for participation. Maternity care providers were sampled based on maximum variation in gender, cadres (see definition) and placement (ward, day/night shift). Maternity care providers could participate in FGDs in phase two, regardless of whether they had been interviewed in phase one or not.

### Participants’ characteristics

The interviewed women were between 15 and 47 years old, with the majority being in their twenties. They included both primi- and multiparous women with different modes of birth and outcomes. The majority had at least received primary education (Table 2). Age of the maternity care providers ranged between 26 and 60 years. The group represented both male and female staff, as well as different cadres, the majority being nurse-midwives (Table 2).

**Table 2:** Characteristics of the participants in the study (women and maternity care providers)

**Women**	**Characteristic**	**Category**	**Participants - individual interviews (n = 23)**	**Participants - FGDs (n = 7)**
	**Age**	15–20	6	0
		21–30	11	3
		31–40	4	4
		≥41	2	0
	**Education level**	No formal education/primary not completed	2	0
		Primary education	14	4
		Secondary or above	7	3
**Maternity care providers**	**Characteristic**	**Category**	**Participants - individual interviews (n = 17)**	**Participants - FGDs (n = 11)**
	**Age**	20–30	8	2
		31–40	4	6
		41–50	1	1
		≥51	4	2
	**Gender**	Female	8	6
		Male	9	5
	**Cadre**	Nurse-midwives	12	7
		Medical doctor or assistant medical officer	5	4

### Analysis

#### Reflexive thematic analysis – from transcripts to themes

All data were analysed and coded using reflexive thematic analysis as described by Braun & Clarke [37, 38]. All transcripts were initially read multiple times in their full-length, during which paragraphs on perceptions of pain management during labour were identified for analysis.

Coding and analysis were data driven, supported by the software MAXQDA, 2022 [39]. Data were analysed by the author KTR with frequent and thorough supervision from FAA. Uncertainties, doubts and questions were addressed, discussed and reflected upon by KTR, FAA and HMA throughout the process.

Themes were generated across the groups of women and maternity care providers. An overview of codes and themes is attached in Appendix A.

## Results

**Fig. 1:**
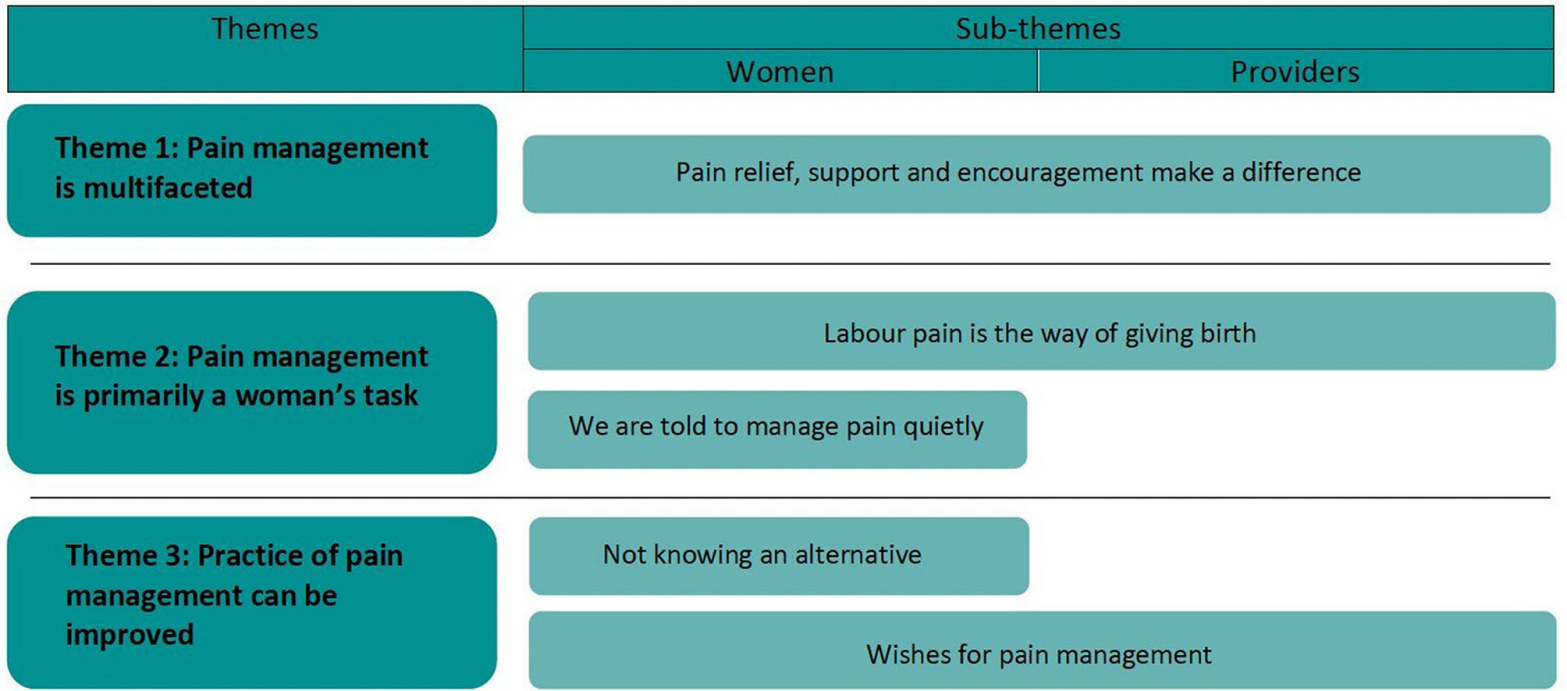
Generated themes and sub-themes

Analysis of all data resulted in three main themes and five sub-themes (Fig. 1).

Theme one and two describe different perceptions of pain management, which appeared to be evident in the maternity care providers’ accounts of their approach to pain management and in women’s experiences and desire for change of pain management practices. Theme three represents a shared perception of pain management as something that can be improved by both women and maternity care providers.

### Theme 1: Pain management is multifaceted

Pain management was described as multifaceted by both women and maternity care providers, involving both pain relief methods and psychological and social support. Maternity care providers identified multiple ways to support and help women through pain and perceived themselves as having a role in utilization of pain management methods to varying degrees. Some women described the use of different pain management methods as important to help them cope with pain during childbirth. Women and providers described both pain relief, support and encouragement as something that could make a difference.

### Pain relief, support and encouragement make a difference

Non-pharmacological pain relief methods (e.g. massage, breathing techniques, position change) were mentioned by both maternity care providers and women as being utilized and as being somehow effective and useful. While some women requested massage repeatedly, for example, a few providers questioned its pain-relieving effect, but recognized that it might still have a comforting effect on women.

Some providers mentioned pharmacological pain relief as a way to manage pain but many, in both hospitals, reported this to be unavailable (despite some availability – as listed in study setting). Few maternity care providers, however, mentioned that it could be available in special rare instances when prescribed by a doctor.

Women perceived support and encouragement provided by maternity care providers as an important aspect of pain management. Some women described that it gave them strength and made them feel safe and more hopeful, thus positively affecting their ability to manage pain during labour. As one woman explained;

*They truly helped me (.) they were saying so sorry sister you will give birth safely, don’t give up, your baby will soon arrive, then I told them I have lost my strength. They said God is there he will help you*” and ”*It helped me a lot - if I was alone, it would not have been easy but because of their company I gained strength” (ID, woman, 16)*

Some maternity care providers recognized that their support could help women to reduce psychological concerns and work better with pain. Several described provision of support and “good language” as the best tools – or as the “main drug” – they had, when helping women to manage pain:

*I see nurses’ words, polite language will help more [than pain relief methods] to console a mother and reduce the struggle on the bed, that is the main drug*” *(ID, maternity care provider, 2)*

While many women experienced and benefitted from support and/or supportive language from their maternity care provider, some women described “good language”, such as saying “pole” (I am sorry), alone was inadequate, since that was “all” some providers did to support them.

### Theme 2: Pain management is primarily a woman’s task

#### Labour pain is the way of giving birth

Although some providers identified different pain management methods and perceived themselves as having a role to play, pain management was commonly perceived as primarily or solely a woman’s task. Such perceptions were linked to maternity care providers who did not demonstrate reflection on what they could do to help women manage pain. Some providers described offering limited to no pain relief and/or support to women during labour. While not all elaborated on why, some implied that methods to manage pain were not necessary as pain is natural and is “the way of giving birth”. Few women shared this perception and directly expressed not seeing any need for pain relief during labour, as they similarly perceived pain as natural and something that would pass fast. One woman explained:

*I would not like it to be [pain being alleviated], because that is how labour pain should be and it fastens the birth process” (ID, women, 7)*

Perceiving pain as natural and necessary was a common theme among maternity care providers, who expressed that they offered limited to no pain relief and/or support. With reference to normalization of labour pain and with the argument that pain will pass once the baby is out, providers described asking women to tolerate pain on their own. Such practice was evident in women’s description of their experiences.

Many women reported not receiving any help to manage pain during labour. They experienced that their pain was normalized or neglected, despite expressing it by screaming, crying and groaning. Some verbalized their pain to the maternity care providers, asking if they understood how much it hurt them. In response, women described being told that pain is part of giving birth and that they should bear with it and persevere. As expressed by a woman who verbalized her pain:

*Eh I told them that it was painful, but they said that is what childbearing is all about*” *(ID, woman, 21)*

Providing limited or no pain relief or support was justified by maternity care providers by describing pain as something God brought, something natural and something all women feel during labour. As one provider explained:

*It happens, there is one mother who asked if there are drugs for relieving pain and I told her that she cannot give birth without pain. To give birth you must experience strong labour pains but she said that ‘No, the pain is very strong’. I told her that it is natural and she wished to go for operation as she thought that there is a drug for releasing labour pains… She forgets that labour pain is the way of giving birth (…).” (ID, maternity care provider, 3)*

Furthermore, as indicated in the quote above, pain was perceived as necessary for the physiological process of childbirth: Necessary for “the baby to come out”, “for the baby to come down” etc. This was in alignment with the belief among some maternity care providers that pharmacological pain relief methods could stop or slow down labour and thereby make it difficult (or not possible) to give birth. As expressed by a provider:

*I just tell her that I cannot give you [anything] to relieve pain because the outcome of that pain is a baby, so when I give you drugs you cannot give birth, so please tolerate because you cannot do it without pain (ID, maternity care provider, 7)*.

In addition, other providers expressed doubts about the effectiveness of using pharmacological pain relief methods and some described not being able to do anything to relieve pain, since no medication was available.

### We are told to manage pain quietly

Besides receiving limited or no pain relief and/or support, women were told to manage their pain quietly. Some women experienced that their expression of pain led to negative communication and collaboration with the maternity care providers. They emphasized especially that making “too much” noise could lead to negative feedback.

*(…) the doctor left, the pain only worsened I was groaning to the point a nurse became angry and reproach completely (…) If you call them [they say] “I’m coming “ - “you go on, just shut up and stop crying, just take a breath, why are you crying? “” (ID, woman, 9)*

Moreover, women expressed that maternity care providers “motivated” them to follow instructions (e.g. to be quiet, do what they say, breathe) by informing them that their behaviour could “jeopardize” the safety of their baby. One woman explained that she was told, she could lose her baby if she disturbed too much and another woman said:

*I was needed to breathe in (…) I should breathe in so that the baby gets to breathe too, if I will be talking while labour pains persist I will be depriving the baby of oxygen (ID, woman, 7)*

No provider directly described a practice where their response to women’s pain was to tell them to be quiet. It was mentioned, however, that the way women expressed their pain, could affect the way a provider might be perceived by others. One provider explained:

*(…) when the child is about to come out, you [a woman in labour] scream loud like the nurse is killing you until other people out there may think of you differently, like “what is that nurse doing with the patients?”. It becomes a challenge” (ID, maternity care providers, FGD)*

### Theme 3: practice of pain management can be improved

#### Not knowing an alternative

While women’s accounts demonstrated that some had knowledge about pain management methods, others expressed that they did not know or had never heard of ways to relieve pain during labour. Many women did not question current practice of pain management or expressed any wish for an alternative, preferred practice. Some women explained “*we have never seen” or “we have never heard” [of ways to relieve pain].* One said:

*Hmmm, there is nothing [you can do to relieve pain], you will have to wait until the baby is out*” *(ID, woman, 16)*

Women reported that they only received information and advice from relatives or their community with regards to coping with labour pains, not from their health care providers in the antenatal care clinic. These accounts did not, however, include advice on how to manage or relieve pain. Instead they were told that labour is painful and “*giving birth is not easy*” and were advised to “*go see for yourself”*. Some women were told by their relatives not to make any noise during labour and to follow instructions from maternity care providers. As a result of not knowing ways to manage pain, some women expressed that they were unsure about what to do once labour started.

Some maternity care providers recognized that many women, especially primiparas, did not have much knowledge about labour prior to childbirth and due to this identified a need for antenatal education. One provider stated that “*education is medicine”* and explained that educating women included describing why they are feeling pain which would help them to better manage it. Providing education and giving information to women during labour was, to a certain extent, described as part of current practice and it was anticipated that general antenatal education could improve women’s preparedness and thus ease collaboration and communication in the labour ward.

### Wishes for pain management

Some women expressed a wish for change of pain management practices. Due to experiencing labour pain as *“severe”*, “*intense*”, “*too much*” and “*excruciating*”, some women expressed a wish for (further) access to pain relief methods - both non-pharmacological and pharmacological. Furthermore, some women would have appreciated continuous support from providers as this made them feel safe (vs. fearful) and helped them to feel better, despite feeling pain. As one woman described:

*I wished the nurse won’t go out I wished she stays there to assist me all the time. The act of calling her all the time to come is what angers them [nurses], if they are impatient and begin to talk badly thinking that you are exaggerating matters, not knowing that you feel safe with her presence*” *(ID, woman, 16)*

Furthermore, women vocalized a wish for comforting language and to be talked to without shouting and to be seen (vs. ignored). Such language, support and encouragement could come from maternity care providers, but also from the companions (who were not allowed into the maternity ward, however), that some women wished to have with them during labour.

Like some women, some maternity care providers expressed a wish for changed pain management practices. No providers, however, expressed something they could do themselves in order to improve pain management. Instead, contextual factors such as shortage of staff and poor ward infrastructure were described as barriers to change, as they hindered the possibility of providing continuous support and including companions during labour. With regards to shortage of staff one provider explained:

*The difference between providing care, in the presence of one mother of course is that you have more time with her there, now with so many mothers you will do this for her, maybe you have not finished another one needs something, so sometimes there is lack of care (…)” (ID, maternity care provider, 10)*

While some providers recognized the benefits of allowing companions in the labour ward, others considered them to be unnecessary and “in the way”. Allowing only some women to be accompanied was not perceived as a possibility, as it would look as though the ward is favouring some women over others.

Also, while some women wished access to pharmacological pain relief, no providers explicitly expressed dissatisfaction with the lack of its utilization in the hospitals. Some providers, however, did express being willing to learn more about its use.

## Discussion

In this study, both women and maternity care providers had various perceptions of pain management, varying from recognizing pain management as being multifaceted to perceiving it as a women’s task to manage pain on their own. Some women considered the current practice of pain management as satisfactory, while others expressed their experiences neutrally and demonstrated limited or no knowledge about alternative practices. Both groups had more or less agreed that current pain management practices could be improved.

Maternity care providers had different perceptions of pain management, in line with other research, reporting large variations in approaches and utilization of pain relief methods and/or support among maternity care providers [20, 22, 40, 41]. The *“pain relief*” and *“working with pain”-approach*, as described by Leap and Hunter, are two commonly adopted approaches to pain management [4]. Whilst these approaches distinguish between *how* to approach pain management, our findings indicated, however, that some providers still question *if* they are, or should be, involved in pain management. This finding highlights the wide range of perceptions among maternity care providers, which may not only affect how pain management is practiced, but also how potential interventions for improvement could be addressed.

Maternity care providers in our study perceived pain management as a multifaceted task. Such perceptions were linked with their accounts for utilizing and referring to not just one, but different pain management methods that addressed both physiological (e.g. utilizing pain relief methods) and psychological and social aspects of pain management (e.g. providing support, comforting language). While this study did not seek to establish why some providers utilize pain management methods and others do not, previous studies found that positive attitudes towards pain management methods, high education level and inner motivation among providers were positively associated with the use of pain management methods [22, 41, 42]. A linkage between educational level and utilization of pain management methods is interesting to consider in further research, especially in a setting like Tanzania where health care staff have multiple levels of training [33, 34].

Some maternity care providers perceived their own role in pain management as limited and thus offered limited or no pain relief and/or support during labour. Such practice is in strong contrast to WHO’s recommendations for intrapartum care, in which pain management and pain relief methods are described as key elements in the provision of good quality care to women during labour [9]. Nonetheless, our findings are similar to previous research from Tanzania and sub-Saharan Africa [20, 23, 24, 26, 43]. One explanation for offering limited or no pain relief and/or support during labour was that some providers perceived pain as natural and necessary and thus found pain management unnecessary. Similar findings are reported in multiple other studies [20–22, 40]. Furthermore, in this study, some maternity care providers had misconceptions about pain being necessary for giving birth and about pharmacological pain relief stopping labour. Similar findings are described in previous studies, where providers had concerns about both non-pharmacological and pharmacological pain relief causing complications, delay in labour progress and having adverse effects on the baby [21, 22]. Such beliefs and reasoning may help to understand why maternity care providers did not explicitly express that access to and usage of pharmacological pain relief would improve the practice of pain management. It may also help explain why providers did not utilize or even mention pharmacological pain relief methods that were already available in the hospitals.

Some women experienced lack of empathy, lack of continuity of care and negative communication and collaboration with maternity care providers during labour pain. Sadly, such experiences of disrespectful maternity care are commonly reported in both Tanzania and in similar contexts where it has been shown to affect women’s experiences and health care seeking behaviour [24, 26, 28, 43, 44]. Women in this study did not explicitly express dissatisfaction due to lack of pain relief methods offered and/or available, however, some women expressed positive attitudes towards utilizing them. On one hand, this study therefore indicates that women’s dissatisfaction might be more closely linked to insufficient and disrespectful care from maternity care providers than to lack of pain relief methods. This is in line with research showing that support from providers and the quality of the woman-provider relationship are some of the main factors influencing women’s experiences during childbirth [8, 14, 15]. On the other hand, one should remember that women expressed positive attitudes towards utilization of pain relief methods, thus expressing a desire for more relief.

In line with previous research, some women demonstrated low or no knowledge about pain management methods, which may lead to inadequate requests for these and challenge women’s participation in decision-making during labour [11, 45]. Being part of decision-making during labour is an important factor influencing women’s experiences and such participation requires knowledge and antenatal education [15, 46]. Some women in our study were told by their relatives to be quiet during labour. In other contexts, cultural perceptions and beliefs about pain have led to women concealing pain during labour, resulting in inadequate demand for pain relief [23, 47]. This indicates that women’s needs are affected by factors such as culture, environment and family and calls for a debate on how to address these aspects.

### Methodological considerations – strengths and limitations

To the best of our knowledge, this is the first study to explore perceptions on pain management during labour of both women and maternity care providers in the same setting in Tanzania. Qualitative interviews, a large sample size and purposive maximum variation sampling ensured involvement of a broad range of both women and maternity care providers who all contributed to an understanding of pain management perceptions and thereby increasing the transferability of the results. Furthermore, our study is strengthened by triangulation, as both individual interviews and respondent checks in the form of FGDs were included.

A limitation of our study is that community members, including fathers, were not included. Also, the interviews explored multiple topics besides pain management. In-depth interviews on pain management only, could have added further points of views and further insights. Another limitation is that the participating women in the FGDs (phase two) were not the same as those in the individual interviews in phase one. Similarly, not all maternity care providers from the individual interviews, participated in the FGD’s. Interviews were conducted in a hospital setting which could have affected how participants reported on their experiences and perceptions. For example, women could have been worried about negative effects on their ongoing care and providers might report more “desirable practice” than reality is. To minimize social desirability bias, all interviews were conducted in a setting that ensured privacy and confidentiality. It is, however, still possible that negative experiences and “less acceptable” views were underrepresented, as some participants might be more comfortable reporting this in a community setting. Such examples were found in other studies, where community follow up interviews showed lower satisfaction, compared to initial exit interviews on discharge [25, 48]. Furthermore, one should consider that women were interviewed relatively shortly after giving birth, which potentially could affect how they experienced childbirth. While one can argue that women’s recall bias must be minimal when being interviewed shortly after giving birth, some women might feel an instant gratefulness and relief of going through labour safely, overshadowing any dissatisfaction with other aspects of labour.

### Implications

Our findings add to the global focus on facilitating positive birth experiences among women, by providing insight into pain management perceptions and practices. It is necessary to address interventions towards both maternity care providers, women and community members to improve pain management. In order to accommodate what women desire for pain management there is a need to increase access to pain relief methods and facilitate continuous care from a provider and/or a companion during childbirth. Additionally, comforting communication was highlighted as important for women to feel safe while experiencing labour pains. Interventions could also focus on education, increasing knowledge and awareness of pain management methods, reducing misconceptions and increasing women’s autonomy. Furthermore, contextual factors hindering pain management practices, such as not allowing companions to be present at actual childbirth, should also be addressed.

## Conclusion

Maternity care providers’ and women’s perceptions ranged from perceiving pain management as involving a combination of physiological, psychological and social aspects to perceiving it as involving limited or no pain relief and/or support. Utilization of pain management was experienced and perceived as both useful and unnecessary. Pain management was commonly perceived by providers as primarily (or solely) women’s responsibility, which led to feelings of women left alone and unsure about how to cope with pain during labour. While some women had similar perceptions of pain management as providers, others expressed a dissonance between the practice they experienced and the practice they would have preferred.

Suboptimal pain management was recognized by some providers who identified contextual factors as a barrier to change pain management practices.

Efforts should be made to improve the current practice of pain management in Tanzania, in order to ensure women’s access to respectful maternity care. We believe this study can help guide the development of future interventions that aim to promote good quality pain management in Tanzania, which if successful, can be beneficial in other, similar contexts.

### Electronic supplementary material

Below is the link to the electronic supplementary material.

Supplementary Material 1

## Data Availability

The datasets of the current study are not publicly available due to the following three principles *”1) qualitative data are difficult to interpret or reuse without extensive knowledge of the context and how is was originally collected (i.e. experience and background of the data collector), 2) with the small number of hospitals included in the project it would be difficult to guarantee participants complete anonymity, and 3) the quality of the response may differ if participants knew their spoken word would be made available in an open repository*” [30], but are available from the corresponding author on reasonable request. Furthermore, our ethics approval were granted based on the anonymity of the individuals who consented to participate.

## Declarations

### Authors? information

The first author of this study is a midwife with previous work experience from Denmark and West Africa. All background information and contextual understanding of Tanzania is based on extensive literature review and collaboration, supervision and discussions with the local research team based in Tanzania. Furthermore, the author had the chance to visit Tanzania and the two study sites during a one-month period during the analysis process to facilitate better contextualization and discussion. All authors have contextual knowledge of Tanzania and have worked extensively in the country.

## Abbreviations

ALERT: Action Leveraging Evidence to reduce perinatal mortality and morbidity in Sub-Saharan Africa
FGD: Focus Group Discussion
LMICs: Low-and Middle-Income Countries
WHO: World Health Organization
