# Supplementary material for: Women’s and maternity care providers’ perceptions of pain management during childbirth in hospitals in Southern Tanzania
Source: BMC Pregnancy Childbirth. 2024 Jun 10;24:417. doi: 10.1186/s12884-024-06606-9 (PMC11163787; doi:10.1186/s12884-024-06606-9)
Supplement: Supplementary file 1 — Supplementary Material 1 [file 12884_2024_6606_MOESM1_ESM.docx]

| Themes  Appendix 1: Generated themes, sub-themes and codes. | Sub-themes | Codes |
| --- | --- | --- |
| 1. Pain management is multifaceted | Pain relief, support and encouragement make a difference | - Pain is an indicator of need of help - Knowledge on non-pharmacological pain relief methods - Knowledge on pharmacological pain relief methods - Advice on how to manage pain - No pharmacological pain relief methods are available - I do not know of pain relief methods - Provider do not believe massage till ease pain - Received advice on how to manage pain - Stress and fear can affect pain - Supportive language can help women manage pain - Receiving supportive language from provider helps - Education and explanation on pain psychological support by providers |
| 2. Pain management is primarily a woman’s task | Labour pain is the way of giving birth | - Pain is a natural thing - Everyone feels labour pain - Provider do not believe medication will help against pain - Pain medication will stop or slow labour - Advice on / approach on how to manage pain - No pain relief or support was offered - We have to bear with the pain - Pain is necessary to give birth |
|  | We are told to manage pain quietly | - Accounts for providers reaction to verbalization of pain - Experience of pain - Expression of pain - We are told to persevere |
| 3. Practice of pain management can be improved | Not knowing an alternative | - Lack of knowledge on pain management methods - Accounts of what they have heard about pain and management prior to delivery - Told not to make noise during labour pain - Do not know what to do about pain |
|  | Wishes for pain management | - Companion can provide care if present (vs. companion will not help much) - Wishing for provider to be present - Not asking for pain relief/help despite wish - Accounts for providers reaction to verbalization of pain - Attitudes towards pain relief - Attitudes and acceptance of medicine if offered - Provider should come when we are in pain - Experience of pain as severe - Pain gives anxiety and fear - Knowledge on pain relief and management - Shortage of staff as a barrier to support - Infrastructure as a barrier to allow companions inside |

Appendix A illustrates an overview of the codes, sub-themes and themes generated in the analysis process of all individual interviews and focus group discussion, with both women and maternity care providers. The table aims to illustrate the analysis process from codes to sub-themes and finally to themes.
